# Multidimensional Assessment of Individuals with Parkinson’s Disease: Development and Structure Validation of a Self-Assessment Questionnaire

**DOI:** 10.3390/healthcare10101823

**Published:** 2022-09-21

**Authors:** Luanne Cardoso Mendes, Isabela Alves Marques, Camille Marques Alves, Marcus Fraga Vieira, Edgard Afonso Lamounier Júnior, Adriano Alves Pereira, Eduardo Lázaro Martins Naves, Fábio Henrique Monteiro Oliveira, Guy Bourhis, Pierre Pino, Yann Morère, Adriano de Oliveira Andrade

**Affiliations:** 1 Centre for Innovation and Technology Assessment in Health (NIATS), Faculty of Electrical Engineering, Federal University of Uberlândia, Minas Gerais 38400-902, Brazil; 2 Laboratoire de Conception, d’Optimisation et de Modélisation des Systèmes (LCOMS), Université de Lorraine, 57070 Metz, France; 3 Assistive Technology Laboratory, Faculty of Electrical Engineering (NTA), Federal University of Uberlândia, Minas Gerais 38400-902, Brazil; 4 Bioengineering and Biomechanics Laboratory (Labioeng), Federal University of Goiás, Goiânia 74690-900, Brazil; 5 Computer Graphics Laboratory (CG), Faculty of Electrical Engineering, Federal University of Uberlândia, Minas Gerais 38400-902, Brazil; 6 Federal Institute of Education, Science and Technology of Brasília—Brasília Campus (IFB), Brasília 70830-450, Brazil

**Keywords:** Parkinson’s disease, remote evaluation, self-assessment, multidimensional assessment questionnaire, patient monitoring, disease progression

## Abstract

(1) Background: Several instruments are used to assess individuals with Parkinson’s disease (PD). However, most instruments necessitate the physical presence of a clinician for evaluation, were not designed for PD, nor validated for remote application. (2) Objectives: To develop and validate a self-assessment questionnaire that can be used remotely, and to assess the respondents’ health condition. (3) Methods: A questionnaire, so-called Multidimensional Assessment Questionnaire for Individuals with PD (MAQPD), was developed, administered remotely, and completed by 302 people with PD. MAQPD was validated using factor analysis (FA). The participants’ level of impairment was estimated using factor loadings. The scale’s accuracy was assessed estimating floor and ceiling effects and Cronbach’s alpha. (4) Results: FA suggested classifying the questions into daily activities, cognition, and pain. The respondents did not have extremely severe impairment (most scores ranged from 100 to 180 points), and the factors with the lowest scores were cognition and pain. The instrument had no significant floor or ceiling effects (rates less than 15%), and the Cronbach’s alpha value was larger than 0.90. (5) Conclusion: MAQPD is the only remote self-administered tool found in the literature capable of providing a detailed assessment of the general health status of individuals with PD.

## 1. Introduction

Parkinson’s disease (PD) is a complex and progressive neurological disorder that causes tremor, rigidity, bradykinesia, and postural instability [1]. PD has the potential to affect an individual on multiple levels, including motor, cognitive, social, and/or emotional aspects [2]. As a result, individuals with PD should be evaluated on a regular basis [3].

Questionnaires are effective tools for multidimensional evaluation [4]. They offer respondents the opportunity to reflect on themselves and provide information that contributes to a better understanding of their health status [5]. Other advantages include: Relatively low costs for both instrument development and data collection; the potential to be answered by a large number of people because they can be applied in person or remotely; no need for an interviewer; temporal flexibility for data collection [6]; and the ability to measure and discriminate the actual needs of individuals, allowing for the development of new technologies designed to solve real problems [7].

Several instruments are being developed to assess general aspects of health and quality of life in people with PD. However, widely used questionnaires and scales, such as the Movement Disorder Society—Unified Parkinson’s Disease Rating Scale (MDS-UPDRS) [8], cannot be conducted remotely because they require the physical presence of a clinician [9]. Furthermore, in a pandemic situation, remote assessment would be useful for disease prevention, assessment, and patient condition monitoring.

The Parkinson’s Disease Questionnaire-39 (PDQ-39) [10] is another widely used questionnaire that has limitations in evaluating some aspects, such as pain, which is a symptom closely linked to quality of life and is present in approximately 85% of people with PD [11]. Since PDQ-39 contains only 39 items, it may not favor a more in-depth assessment, particularly if used remotely.

The Barthel Index [12] and Katz Index [13] are two other scales used to assess people with PD. Nonetheless, aside from not evaluating different aspects, neither scale is specific for PD. The Barthel Index was created to assess post-stroke individuals [14], and the Katz Index to assess elderly people [13]. Therefore, using these scales to assess people with PD may result in inaccurate assessments.

In summary, most of the instruments used to assess individuals with PD have the following limitations: they require the physical presence of a clinician to perform the assessment, were not designed specifically for patients with PD, and/or have not been validated to be self-administered or applied remotely. As a result, the need for developing a self-applied instrument capable of being used remotely, which can provide a detailed assessment of the overall health status of people with PD, was identified.

The goals of this study were to create and validate the structure of a multidimensional self-assessment questionnaire for people with PD that can be used remotely, to propose a model for calculating the final score and level of impairment of the respondents, to assess the scale’s accuracy, and to identify the most common limitations of individuals with PD.

## 2. Materials and Methods

### 2.1. Questionnaire Development

The questionnaire’s development and application were approved by the Ethics Committee for Human Research at the Federal University of Uberlandia.

Validated questionnaires and assessment scales such as MDS-UPDRS [8], PDQ-39 [10], Barthel Index [12], Katz Index [13], and Multidimensional Pain Inventory (MPI) [15] were used in the development of the instrument. Food, Mobility, Sanitation, Clothes, Miscellaneous, Memory/Concentration, Social and family conviviality, Personality, Energy, Emotion/Humor, Severity of pain, Interference, Perceived life control, and State of mood-affectivity were created as subgroups of questions.

The “Multidimensional Assessment Questionnaire for Individuals with Parkinson’s Disease” (MAQPD) questionnaire was proposed, with a total of 83 evaluation questions (Appendix A). The language of the questionnaire was Portuguese, it was created using Google Forms (a survey administration software), and it was applied to Portuguese speakers from Brazil. MAQPD was divided into three sections:

1. Section A—Research presentation. This section presents the aims of the research, describes the risks and benefits, as well as the importance of the participation of people with PD.
2. Section B—Questions about daily activities (Module 1), related to self-care and the capacity of individuals to live independently; and questions concerning cognitive aspects (Module 2), related to the individuals’ feelings about life and society. This section presents 66 questions for evaluation by the respondents, of which 43 are distributed to the subgroups of Module 1 (7 for Food, 12 for Mobility, 7 for Sanitation, 7 for Clothes and 10 for Miscellaneous), and 23 distributed to the subgroups of Module 2 (2 for Memory/Concentration, 6 for Social and family conviviality, 3 for Personality, 6 for Energy and 6 for Emotion/Humor).
3. Section C—Multidimensional Pain Inventory. This section presents four subgroups of questions taken from the MPI (Portuguese version) [15], which refer to the impact of pain on the life of individuals. In this section there are 17 questions for assessment by the respondent, of which 3 are for the subgroup Severity of pain, 9 for Interference, 2 for Perceived life control, and 3 for State of mood-affectivity.

MAQPD uses two response scales: a LIKERT scale with five classification levels (0—Totally disagree; 1—Disagree; 2—Neutral; 3—Agree; and 4—Totally agree) used in Section B, and a LIKERT scale with seven classification levels ranging from 1 (little pain) to 7 (a lot of pain) used in Section C. In addition, the questionnaire presents optional open-ended questions so that respondents have the opportunity to express in detail complaints or aspects not mentioned in the questionnaire. The average time it took to respond to MAQPD was 18 min.

### 2.2. Questionnaire’s Structure Validation

#### 2.2.1. Research Participants

People with PD were invited to participate in the study through a variety of channels, including email lists, discussion forums, PD webinars, interviews for local television stations, and WhatsApp groups. The inclusion criteria for the study were: Having a confirmed diagnosis of PD from a neurologist or geriatrician; and being able to fill out the questionnaire independently. Individuals whose responses to the questionnaire were incomplete were excluded from the analysis. MAQPD was completed by 310 people, but 8 of them did not have PD. As a result, 302 answers were analyzed.

#### 2.2.2. Factor Analysis

Factor analysis (FA) was used to validate the structure of MAQPD. FA is a multivariate statistical method used to define underlying structures in data matrices. FA investigates the interrelationships between multiple variables in order to derive a reduced linear structure from the original data [16]. As a result, in a questionnaire with many variables (83 in this study), FA seeks to group such variables into reduced sets described by factors [17].

In short, FA can be performed in two stages: factor extraction and factor rotation. Factor extraction involves choosing the number of factors to be extracted, and factor rotation aims to obtain a simple structure to improve data interpretability. One approach to factor extraction is principal component analysis (PCA), which explains correlations by explaining (common) variance [17].

PCA analyzes a data set in which observations are described by several quantitative dependent variables that are intercorrelated. The aim of this method is to express important information extracted from the data set as a set of new orthogonal variables. In addition, PCA represents the pattern of similarity of observations and variables [18].

PCA calculates new variables obtained as linear combinations of the original variables, called principal components. The first principal component is required to have the highest possible variance, and in this way, this component explains the largest part of the data set. The second and others components are computed under the restriction to have the biggest variance possible and to be orthogonal to the previous component. The values of these new variables for the observations are called factors scores, which can be interpreted in a geometric way as the observations projections on the principal components. The number of factors retained can be observed using the Scree Plot [17,18].

First, the data were normalized in order to convert the response scale of Section C to the response scale of Section B. The normalization was done to satisfy the following relationship: a low score indicates severe impairment, whereas a high score indicates less impairment. Equation (1) was used to normalize questions 67 to 78 and 81 to 83, as these items have an inverse relationship with the response scale from 0 to 4. Equation (2) was applied to questions 79–80 because they are directly related to the response scale of 0–4. For both equations, *X*1 represents the normalized variable on a scale of 0 to 4, and X2 represents the variable on a scale of 1 to 7.

(1) $$X1=-4\times \left(\frac{-X2+7}{6}\right)$$

(2) $$X1=4\times \left(\frac{X2-1}{6}\right)$$

The Bartlett sphericity and Kaiser–Meyer–Olkin (KMO) tests were used to determine whether the dataset met the requirements of FA. The Scree Plot was used to determine the number of retained factors that were rotated (oblimin) to improve their interpretation and relationship with the original variables.

To improve the overall solution of FA, the following criteria were used to exclude irrelevant variables that satisfied at least one of the three criteria:

1. Variables with factor loadings less than 0.35 were considered insignificant based on the size of a sample between 250 and 349 individuals [17].
2. Variables with very similar factor loadings, i.e., difference between factor loadings less than 0.10, in more than one factor [19].
3. Variables with communality under 0.30 [20,21]. Low communality indicates they are not linearly correlated and, therefore, should not be included in FA.

After excluding variables that did not meet these criteria, FA was performed again to re-evaluate the need for additional variable exclusion.

### 2.3. Data Analysis

#### 2.3.1. Assessment of Respondents’ Level of Impairment

FA assigns a factor loading to each item of the questionnaire, which reflects the importance of the item in representing a given factor. To compute the final score for each respondent, the factor loadings were multiplied by the score given by the respondent in each question, and the sum of these values was calculated.

The Spearman’s correlation coefficient between variables was estimated to determine whether the proposed model is consistent. The correlation coefficient was classified into four categories: very weak (0.00–0.19), weak (0.20–0.39), moderate (0.40–0.59), strong (0.60–0.79), and very strong (0.80–1.00) [22].

The final scores of individuals were analyzed using descriptive statistics such as mean, standard deviation, median, and minimum and maximum values. These measures were computed for the entire instrument as well as for each of the FA-defined factors. The violin plot was shown, which reveals additional details about the data distribution. To gain a better understanding of the distribution of the respondents’ final scores, the histogram and cumulative distribution function of the entire instrument, as well as the measures of skewness and kurtosis, were examined.

#### 2.3.2. Scale Accuracy Assessment

The floor and ceiling effects, as well as Cronbach’s alpha, were used to assess the scale’s precision. When a proportion of respondents achieves the lowest possible score across a given domain, the floor effect occurs, preventing the identification of changes in situations where the health condition deteriorates. The ceiling effect is interpreted in the opposite way as the floor effect [23]. When the floor and ceiling effect rates were less than 15%, they were considered satisfactory [24,25].

Cronbach’s alpha is a coefficient that can be used to determine whether or not the questionnaire items are homogeneous and whether or not the scale consistently measures the characteristic for which it was designed. If it was larger than 0.75 it was deemed adequate [26]. Floor and ceiling effect rates, as well as Cronbach’s alpha, were calculated for the entire instrument and for each domain.

#### 2.3.3. Identification of Motor Difficulties and Limitations Related to Cognition and Pain

The most common answer (i.e., mode) for each question was identified in order to find the basic activities of daily living that the respondents had more difficulty performing, as well as the cognitive and pain aspects most affected in these individuals. The responses from the optional open-ended questions were also evaluated.

In addition, the main motor, cognitive, and pain limitations were identified and discriminated for each group of respondents according to their diagnosis time, also calculating the most common answer for each question.

## 3. Results

### 3.1. Questionnaire Structure’s Validation

As shown in Table 1, the majority of those who responded to the questionnaire (28%) had been diagnosed with PD for more than ten years, while the minority (4%) had been diagnosed for two to four years.

**Figure 1:**
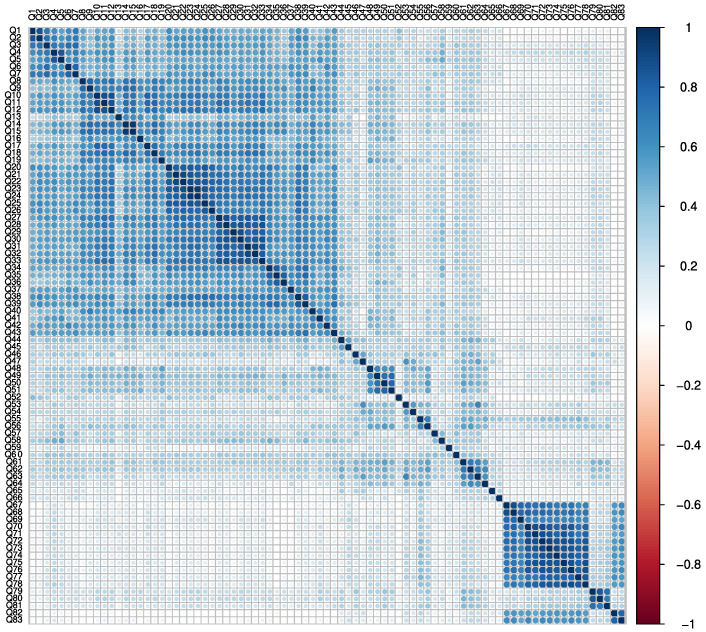
Data correlation matrix before FA.

The adequacy of the data for the application of FA was verified. The results of the Bartlett’s test of sphericity (χ^2^ = 28247.25; df = 3403 degrees of freedom; *p* < 0.001) and KMO test (overall Measure of Sampling Adequacy of 0.95) suggested a good adequacy of the data to FA. Figure 1 shows the data correlation matrix of the questionnaire.

PCA was used to confirm that three factors should be retained because the Scree Plot showed a linear decreasing trend at Component 3.

To improve the interpretation of the factors, the oblique rotation oblimin was used, and the factor loadings and communality were analyzed to eliminate twelve irrelevant variables. Question 45, 52, 57, 59, 66, and 80 had factor loadings less than 0.35; question 44 had factor loadings for two factors that were very similar; and questions 45, 52, 57, 58, 59, 60, 65, 66, 79, 80, and 81 had communalities less than 0.30. After these variables were removed, the questionnaire presented 71 items rather than 83.

**Figure 2:**
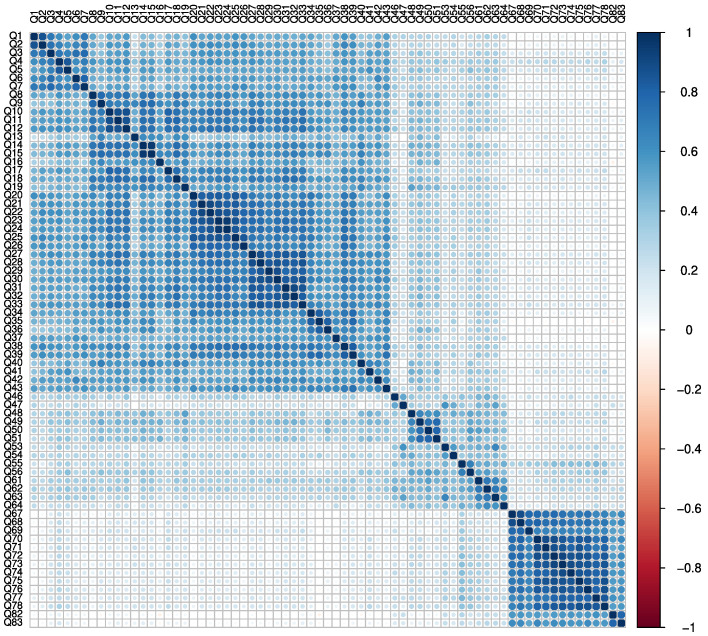
Data correlation matrix after FA. The questions are numbered in accordance with the original questionnaire.

FA was performed once more to ensure that the items in the new MAQPD (Appendix B) met any of the defined variable exclusion criteria. The results showed that the data was adequate for FA, as confirmed by the Bartlett’s test of sphericity (χ^2^ = 26020.98; df = 2485 degrees of freedom; *p* < 0.001) and the KMO test (overall Measure of Sampling Adequacy of 0.95). Figure 2 depicts the new questionnaire’s data correlation matrix.

Three factors should be retained because their proportion of variance explained was larger than 60% [19] and the Scree Plot showed a decreasing linear trend at Component 3.

Oblique rotation oblimin was used to improve the interpretation of the factors, and the variables were reorganized into three different groups:

1. FACTOR 1: Comprised of 43 variables related to the activities of daily living (ADL).
2. FACTOR 2: Comprised of 14 variables related to pain.
3. FACTOR 3: Comprised of 14 variables related to cognitive aspects.

No other variables needed to be excluded.

### 3.2. Assessment of Respondents’ Level of Impairment

The factor loadings of each questionnaire item are shown in Table 2.

The model in Equation (3) was used to compute the sum of each respondent’s final score.

(3) $$Final\;Score=0.57S_{Q1}+0.63S_{Q2}+0.60S_{Q3}+0.57S_{Q4}+0.50S_{Q5}+0.57S_{Q6}+\cdots +0.64S_{Q83}$$

where $S_{Q}$ is the score given by the individual for each question.

**Figure 3:**
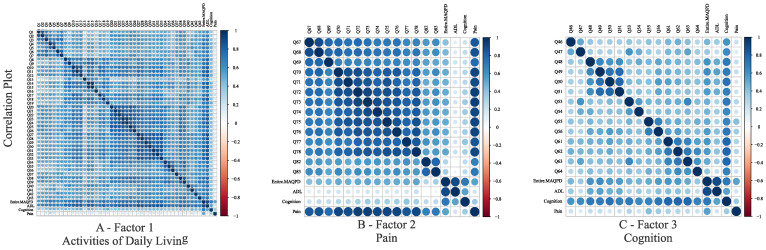
Correlation plots showing the correlation between variables of Factor 1 (**A**), Factor 2 (**B**), and Factor 3 (**C**), and the final scores.

Figure 3 shows the correlation plots for the variables of each factor and the total scores of each domain. The correlation between the total score of the entire questionnaire and the total score of each of the three domains was 0.89 (ADL), 0.54 (Pain), and 0.72 (Cognition).

Individuals’ final scores could range from 0 to 209.36 points if they answered “0” and “4” to all questions, respectively, according to the model. In addition, the lower the score, the more impaired the individual is.

Table 3 shows the descriptive statistics of the final questionnaire scores for the entire questionnaire as well as for each domain.

**Figure 4:**
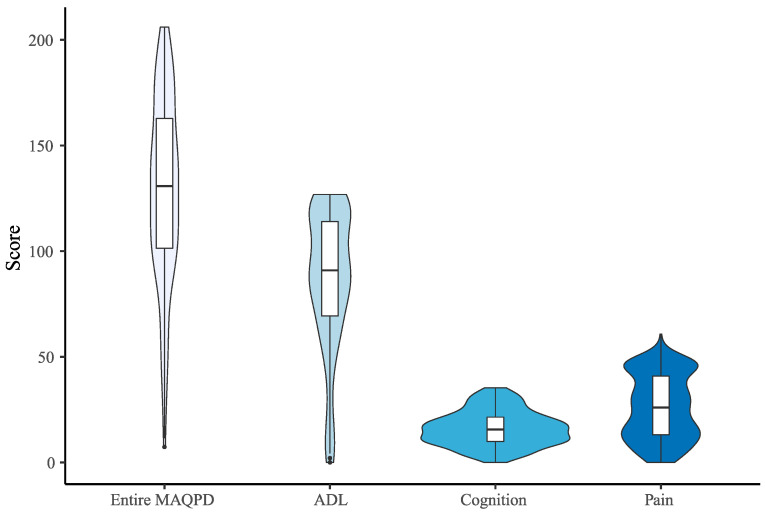
Violin plot of the final scores.

Figure 4 presents the violin plot of the final scores.

**Figure 5:**
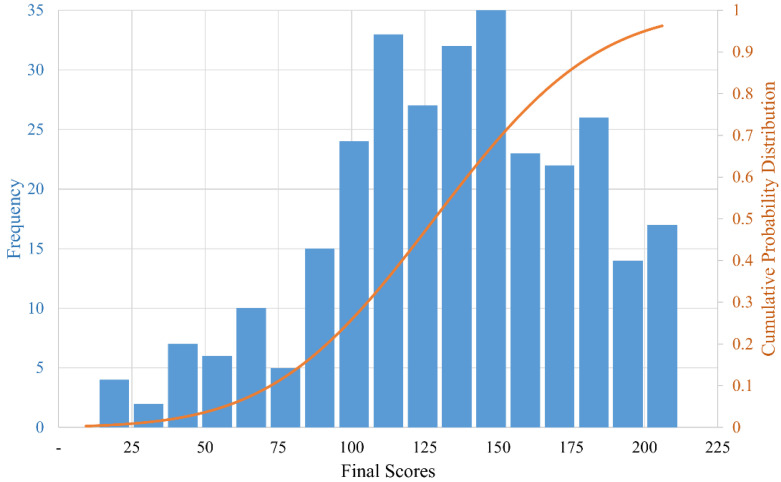
Histogram and cumulative probability distribution function of final scores.

The histogram and cumulative probability distribution function of the individuals’ final scores are depicted in Figure 5.

The frequency distribution of the data is shown by the histogram. Most scores ranged from 100 to 180 points, and only a few people had low scores, i.e., less than 50 points. The cumulative distribution function expresses the likelihood that a random observation sampled from the population will be less than or equal to a given value. The probability of an individual’s final score being between 100 and 180 points was approximately 62 percent, and the probability of the score being less than half of the possible score (approximately 105 points) was approximately 30 percent. The skewness was −0.37 and the kurtosis was −0.30.

### 3.3. Scale Accuracy Assessment

Table 4 shows the estimated floor and ceiling effects of the final scores, as well as Cronbach’s alpha, for the entire questionnaire and for each domain.

There were no significant floor or ceiling effects found, with values ranging from 0.28% to 10.85%. Cronbach’s alpha was larger than 0.90 for all dimensions, indicating that the proposed questionnaire has strong internal consistency.

### 3.4. Identification of Motor Difficulties and Limitations Related to Cognition and Pain

According to the responses, the activities of daily living that people with PD had more difficulty which were represented by Question 13 with a prevalence of zero, indicated that most people completely disagree with the statement; and Question 16 with a prevalence of one indicated that most people disagree with the statement.

The most affected cognitive aspects in patients are represented by the following questions: 48, 49, 50, and 51, which are about living with family and society; 53 and 54, which are about an individual’s personality; 55 and 56, which are about energy and mood; and 63 and 64, which are about emotion and feeling. All of these questions had a score prevalence of one.

In terms of pain, the following questions indicate the most impairment of the individual: 67, 68, 72, 76, and 83. All questions had a prevalence of scores equal to one.

**Figure 6:**
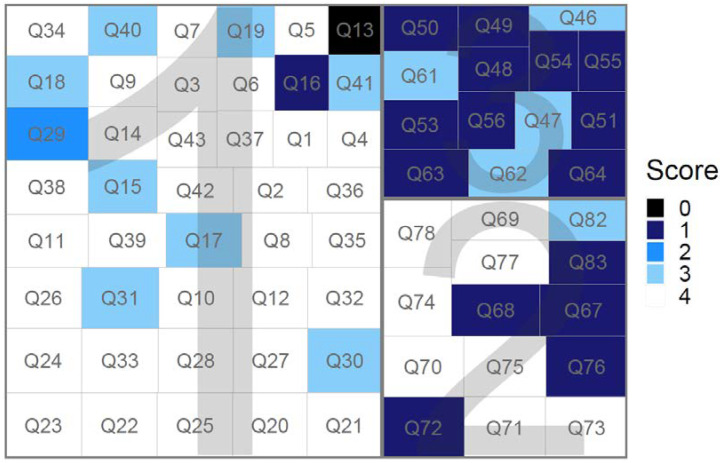
TreeMap of the questionnaire. The colors represent the most common scoring for each question. The higher the factor loading, the larger the area of the rectangle. Each factor has its own set of questions (1, 2 or 3).

Figure 6 depicts the TreeMap of the questionnaire. The color gradient corresponds to the most frequently assigned score for each question. The rectangles’ sizes are proportional to the factor loading of the corresponding question. Each factor has its own set of questions.

Additionally, 41.39% of the respondents answered the open-ended questions. Of these, most reported problems in performing household chores, such as washing dishes, cleaning the house and cooking.

Table 5 shows the questions that most represent motor, cognitive, and pain limitations, discriminated according to the time of diagnosis of the respondents. Only the questions with a score equal to 0 (totally disagree) or 1 (disagree) were considered.

## 4. Discussion

This work was created in response to the lack of an instrument for remotely assessing the health of people with PD. A study [27] emphasized the importance of using MDS-UPDRS to continuously assess individuals with PD, even in situations with limited physical contact. However, no validated alternatives based on questionnaires or scales for remotely assessing individuals with PD have been fully validated so far.

Motolese et al. [28] aimed to monitor individuals with PD remotely, during the social isolation caused by COVID-19. To monitor the cognitive and motor performance of PD patients, Motolese et al. [28] applied five assessment questionnaires and proposed the use of a smartphone application. The five questionnaires totalized 91 items for evaluation that should be answered by the participants. Falla et al. [29] assessed, longitudinally, motor and non-motor aspects using nine structured questionnaires, which resulted in a total of 148 questions.

First, the questionnaires applied by Motolese et al. [28] were not developed and validated to be applied remotely. Furthermore, answering this large amount of questions may be unfeasible—especially if dealing with individuals with neurological disorders—and demands time from both study participants and researchers. In addition, these questionnaires were administered via telephone and remote web-based video calls by the researchers (i.e., they were not self-administered), i.e., participants did not have the opportunity to answer the questions privately. Thus, it is possible to note the importance of using a more concise instrument capable of assessing multidimensional aspects of the disease, which can be answered privately in an online version, and which can be used for remote monitoring of individuals, associated, for example, with the use of wearable sensors. As a result, we proposed developing and validating the structure of an online questionnaire for multidimensional assessment, as well as a model for estimating individuals’ final score, which is related to their level of impairment.

MAQPD was developed on the basis of existing assessment instruments. It was answered by a large number of people with PD, which contributed to a more robust validation of the instrument structure using FA. This method was used to verify each question’s contribution to the multidimensional evaluation and to group questions into latent dimensions, implying a better distribution and arrangement of the questions [16,17]. FA suggested removing 12 questions and categorizing them into three dimensions: ADL, cognition, and pain. The final version of MAQPD was validated for Portuguese speakers, and then it can be used for the remote assessment of individuals with PD in other studies.

The estimate of factor loadings from FA enabled weighing the final scores based on the relevance of the question to define the factor. Correlation coefficients ranging from 0.54 to 0.89 (Figure 3) were estimated between the overall questionnaire’s final score and the scores for each domain, indicating a moderate to very strong correlation represented by the model (Equation (3)). The correlation between the final ADL domain scores and pain, on the other hand, was very weak (0.19). This could have happened because aspects of one domain may not interfere with the other, i.e., a person may have severe motor impairments but not feel pain.

The final scores for the entire MAQPD were higher than the measures for the three domains, as expected, because the instrument contains 71 questions in total, while the subgroups of questions related to ADL, cognition, and pain contained 43, 14, and 14 questions, respectively (Table 3). The relatively large standard deviation found for the scoring of all domains reveals that individuals with varying levels of impairment responded to the questionnaire, which can be explained by the respondents’ varying diagnosis times.

When compared to pain, the scores for cognition questions were lower (Figure 4). This means that the individuals are more susceptible in cognitive aspects than in pain-related aspects. Since the questionnaire was completed during a period of social isolation, many of the respondents may have developed depression and negative feelings about life, society, and the future (Table 5).

The data distribution (Figure 5) reveals that the majority of respondents scored between 100 and 180 points, and that the cumulative distribution function displayed a higher growth (steeper slope) between these values, indicating a high probability (62%) of an individual’s score falling between these two values. These findings point to the absence of severe impairments in the individuals, which may be related to the fact that the individuals with PD were able to complete the questionnaire themselves.

The skewness and kurtosis coefficients of −0.37 and −0.30, respectively, indicate that the final scores had higher values and were dispersed, indicating a sample with lower impairment but not homogeneous (various diagnosis times).

Floor and ceiling effects were not found in statistically significant proportions across the entire instrument or across any domain (Table 4). As a result, MAQPD has the potential to be a tool for measuring the worsening and improvement of a person’s health. The ceiling effect in the ADL domain was relatively high (10.85%). An explanation for this is that the respondents may have answered the questionnaire soon after taking PD medication, which may have given them the false impression that they could perform the activities. Another possible explanation is that the individuals do not have severe motor impairment in comparison to the cognitive aspects. Cronbach’s alpha was higher than 0.90 for all domains, implying the instrument’s high reliability and internal consistency.

Question 13—“I drive without difficulty” was the only one in the whole questionnaire that presented a prevalence of answer equal to zero, meaning that most individuals responded to this statement that they totally disagree. In this case, a physiotherapist can direct a rehabilitation session that focuses on specific exercises for alleviating the symptoms that prevent the patient from performing the activity, i.e., driving. Therapies may improve specific functions, allowing the activity to be performed again by the individuals.

Furthermore, it was determined that approximately 71.43% of the questions pertaining to cognitive aspects had a prevalence of answers equal to one. This indicates that the majority of respondents had issues with social and family coexistence, lacked the energy and motivation to develop routine activities, and struggled to control their emotions (Table 5). It is noted that more research in the areas covered by these questions is required.

Regarding pain, 35.71% of the questions presented prevalence of answers equal to one (a lot of pain), one of them being question 76—“How much did your pain affect your ability to do housework?”. This result is associated with that obtained by the open-ended questions, for which most of the respondents answered that they had difficulties in performing housework, suggesting that one of the reasons why individuals feel difficulties in performing domestic chores is pain.

Table 5 shows the main limitation by the respondents according to their diagnosis time. As the diagnosis time is a concrete information (it is not subjective, such as the degree of disease impairment interpreted by the health professional when applying the MDS-UPDRS) closely associated with greater impairment and worse perceived quality of life [30], progression of motor disability and higher risk of dementia [31], and loss of independence [32], the diagnosis time may be an effective variable to guide specific treatments for individuals with PD.

Individuals with a diagnosis time of less than 2 years, for example, reported that they had great difficulty in dancing, and that they suffered from a variety of problems related to cognitive issues. For these individuals, doing activities that exercise balance, as well as therapy sessions, may be effective ways to solve these problems. In addition, individuals with a diagnosis time between 2 and 4 years reported that they had difficulty in driving and that they did not meet their friends as much as they would like. Possibly, training the body movements that affect driving—either by using a serious game in which the player is stimulated to drive virtually, or with conventional physiotherapy—may contribute toward the individual performing this activity more easily. Furthermore, promoting support groups with social activities in therapy centers, as well as informing friends and family members about the issue related to social interaction, may help to improve the conviviality of these individuals.

Additionally, as individuals with diagnosis time of less than 2 years, between 6 and 8 years and more than 10 years had lack of social interaction, mood changes, and/or lack of disposition, performing a physical activity that requires relating to other people, e.g., dancing, becomes an extremely complex task. These findings agree with those reported by Darweesh et al. [33]. These may be the reasons why these individuals experience difficulties in dancing. Possibly, these individuals did not feel severe pain due to the short diagnosis time of the disease.

A different consideration that also should be highlighted is that all groups presented limitations related to cognitive aspects, which indicates that this domain needs a more attentive and careful look by family members, health professionals, and researchers. In addition, with the exception of the group with diagnosis time of less than 2 years, all the other groups reported problems related to pain. Possibly, improving aspects related to cognition may contribute to reducing the pain experienced by individuals, since one may entail the other.

The results of an evaluation based on multidimensional questionnaires contribute to a comprehensive understanding of the individual’s health status, as well as their true limitations and needs [4,5]. Understanding these characteristics is a critical step in the development of technologies or products that are centered on the needs of individuals.

## 5. Conclusions

A questionnaire, so-called MAQPD, was developed and its structure was validated by using FA, which allowed for grouping the questions into three domains: ADL, cognition, and pain. The final score for each respondent was calculated using the factor loadings as the weight of the questions. The respondents did not present an extremely severe level of impairment. As the instrument did not present substantial floor or ceiling effects, it can measure both the clinical worsening and improvement of the respondents. The ADL that respondents had the most difficulty in performing was driving. Pain and cognition were also relevant aspects.

In conclusion, the MAQPD is the only self-administered questionnaire found in the literature capable of being used remotely, and which can provide a detailed assessment of the general health condition of people with PD.

## Figures and Tables

**Table 1:** Diagnosis time.

Diagnosis Time	Number of Respondents	Percentage of Respondents (%)
less than 2 years	59	19.54
2 to 4 years	13	4.30
4 to 6 years	51	16.89
6 to 8 years	64	21.19
8 to 10 years	29	9.60
more than 10 years	86	28.48

**Table 2:** Factorial loadings of the questionnaire’s 71 items. The questions are numbered in accordance with the original questionnaire.

Variable	Factor Loadings	Variable	Factor Loadings	Variable	Factor Loadings	Variable	Factor Loadings
Q1	0.57	Q19	0.57	Q37	0.59	Q62	0.72
Q2	0.63	Q20	0.91	Q38	0.80	Q63	0.79
Q3	0.60	Q21	0.91	Q39	0.78	Q64	0.72
Q4	0.57	Q22	0.92	Q40	0.65	Q67	0.84
Q5	0.50	Q23	0.94	Q41	0.55	Q68	0.85
Q6	0.57	Q24	0.91	Q42	0.65	Q69	0.75
Q7	0.59	Q25	0.92	Q43	0.62	Q70	0.92
Q8	0.71	Q26	0.87	Q46	0.48	Q71	0.93
Q9	0.65	Q27	0.88	Q47	0.60	Q72	0.94
Q10	0.84	Q28	0.89	Q48	0.60	Q73	0.93
Q11	0.83	Q29	0.82	Q49	0.56	Q74	0.89
Q12	0.84	Q30	0.88	Q50	0.66	Q75	0.92
Q13	0.50	Q31	0.87	Q51	0.60	Q76	0.91
Q14	0.67	Q32	0.84	Q53	0.69	Q77	0.80
Q15	0.68	Q33	0.91	Q54	0.55	Q78	0.90
Q16	0.60	Q34	0.80	Q55	0.55	Q82	0.61
Q17	0.76	Q35	0.70	Q56	0.61	Q83	0.64
Q18	0.80	Q36	0.63	Q61	0.69		

**Table 3:** Descriptive analysis of MAQPD scores.

Domain	Mean (Sd)	Median	Min–Max
Entire MAQPD (range: 0–209.36)	128.25 (43.60)	130.78	7.31–206.36
ADL (range: 0–126.84)	86.21 (32.91)	90.91	0–126.84
Cognition (range: 0–35.24)	16.16 (8.25)	15.57	0–35.24
Pain (range: 0–47.28)	25.87 (15.03)	25.71	0–47.28

**Table 4:** Analysis of the accuracy of the MAQPD scale.

Domain	Floor Effect (%)	Ceiling Effect (%)	Cronbach’s Alpha
Entire MAQPD	0.28	3.77	0.9767
ADL	0.44	10.85	0.9839
Cognition	2.50	1.04	0.9270
Pain	4.32	8.2	0.9736

**Table 5:** Main motor, cognitive, and pain limitations of the groups defined by the diagnosis time of the disease.

Diagnosis Time	Main Limitations—Question (Score) *		
	ADL	Cognition	Pain
Less than 2 years	Difficulty in dancing—Q16 (0)	Lack of social interaction and leisure —Q48 (1), Q49 (1), Q50 (1), Q51 (1) Mood changes—Q53 (1), Q54 (1) Lack of disposition—Q55 (1) Lack of happiness and future prospects—Q61 (1), Q62 (1), Q64 (1)	none
2 to 4 years	none	Mood changes—Q54 (1) Lack of disposition—Q55 (1) Lack of future prospects—Q64 (1)	Experiencing severe chronic pain—Q67 (1), Q68 (1) Interference of pain in different aspects of life (e.g., participation in social and leisure activities, ability to do housework)—Q70 (1), Q71 (1), Q72 (1), Q73 (1), Q75 (1) Mood changes—Q82 (1), Q83 (1)
4 to 6 years	Difficulty in driving—Q13 (0)	Lack of social interaction—Q51 (0)	none
6 to 8 years	Difficulty in driving—Q13 (0) Difficulty in dancing—Q16 (0)	Lack of social interaction—Q49 (0), Q51 (0)	Experiencing severe chronic pain—Q67 (1), Q68 (1) Interference of pain in different aspects of life (e.g., participation in social and leisure activities, ability to do housework)—Q70 (1), Q71 (1), Q72 (1), Q73 (1), Q75 (1), Q76 (1) Mood changes—Q83 (1)
8 to 10 years	Difficulty in walking—Q9 (1), Q19 (1) Difficulty in dressing oneself (loss of independence)—Q28 (1) Difficulty in holding a child—Q40 (1)	Lack of social interaction and leisure—Q48 (1), Q49 (1), Q50 (1), Q51 (1) Mood changes—Q53 (1), Q54 (1) Lack of disposition—Q55 (1), Q56 (1) Lack of future prospects—Q64 (1)	Experiencing severe chronic pain—Q67 (1), Q68 (1), Q69 (1) Mood changes—Q83 (1)
More than 10 years	Difficulty in driving—Q13 (0) Difficulty in dancing—Q16 (0)	Lack of social interaction and leisure—Q48 (1), Q49 (1), Q50 (1), Q51 (1) Mood changes—Q53 (1) Lack of disposition—Q55 (1), Q56 (1) Lack of happiness and future prospects—Q61 (1), Q64 (1)	Interference of pain in different aspects of life (e.g., participation in job, ability to do housework)—Q75 (0), Q76 (0)

* To simplify the interpretation and summarize the amount of information present in Table 5, when a given factor presented at least one question with a score equal to 0, the questions of the same factor with a score equal to 1 were omitted from the table.

## Data Availability

The datasets generated in the current study are not publicly available due to the ethical restrictions preventing public sharing of data. A non-identified set may be requested after approval from the Review Board of the Institution. Requests for the data may be sent to the corresponding author.

## Abbreviations

PD: Parkinson’s disease; MDS-UPDRS: Movement Disorder Society—Unified Parkinson’s Disease Rating Scale; PDQ-39: Parkinson’s Disease Questionnaire-39; MPI: Multidimensional Pain Inventory; MAQPD: Multidimensional Assessment Questionnaire for Individuals with Parkinson’s Disease; FA: factor analysis; KMO: Kaiser-Meyer-Olkin; PCA: Principal Component Analysis; ADL: activities of daily living; COVID-19: Coronavirus disease.

## Appendix A. Multidimensional Assessment Questionnaire of Individuals with Parkinson’s Disease

Section A—Presentation of the Survey and Sample Description
I declare that I have Parkinson’s disease and that I am able to answer the questionnaire. *
( ) Yes
( ) No

I was diagnosed with Parkinson’s disease about: *
( ) Less than 2 years
( ) 2 to 4 years
( ) 4 to 6 years
( ) 6 to 8 years
( ) 8 to 10 years
( ) More than 10 years

The symbol * indicates that the participant must answer the question.

Section B—Individual Perception about Parkinson’s Disease
The following statements refer to some aspects of your daily life. Please indicate how much you agree or disagree with each statement. To do this, please answer with one of the five rating levels below:
0—Totally disagree
1—Disagree
2—Indifferent
3—Agree
4—Totally Agree

MODULE 1—Activities of daily living (ADL)
This module refers to activities of daily living, that is, tasks related to self-care and your ability to live independently.

Food

(1) I hold the flatware without assistance.

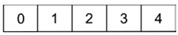

(2) I handle the flatware without assistance.

(3) I take the flatware with the food up to my mouth without help and without spilling it.

(4) I chew the food without difficulty.

(5) I have no difficulty swallowing food.

(6) I serve liquid drinks without help and without spilling.

(7) I drink liquids without help and without spilling.

Mobility

(8) I walk short distances without help.

(9) I walk long distances without help.

(10) I lie in bed without help.

(11) I get up from bed without help.

(12) I sit without help.

(13) I drive without difficulty.

(14) I climb stairs without help.

(15) I go down stairs without help.

(16) I dance without difficulty.

(17) I turn to bed at night without help.

(18) I get up from the car seat without help.

(19) I have balance when walking.

Sanitation

(20) I turn on the shower without help.

(21) I wash my body without help.

(22) I dry myself with the towel without help.

(23) I sit on the toilet without help.

(24) I get up from the toilet without help.

(25) I clean myself after evacuating without help.

(26) I brush my teeth without help.

Clothes

(27) I wear a pant/skirt without help.

(28) I wear a shirt/blouse without help.

(29) I button my clothes without help.

(30) I open/close the zipper of my clothes without help.

(31) I put on my shoes without help.

(32) I tie the shoelaces without help.

(33) I take off my clothes to shower without help.

Miscellaneous

(34) I take my medicines without help.

(35) I make phone calls without help.

(36) I type messages on my cell phone without help.

(37) I write on a sheet of paper without help.

(38) I comb my hair without help.

(39) I use the television remote control without assistance.

(40) I hold a child in my arms without help.

(41) I speak clearly and without difficulty.

(42) I use Gillette without cutting myself.

(43) I read texts without help.

Is there any other activity in your daily life that you have a lot of difficulty or desire to do that has not been mentioned in this questionnaire?

MODULE 2—Cognitive aspects
This module refers to the cognitive aspects, that is, your feelings about life and society.

Memory/Concentration

(44) I concentrate to accomplish the tasks.

(45) I remember things without having to write them down.

Social and family conviviality

(46) I feel close and welcomed by other people.

(47) I am calm with other people.

(48) My physical condition does not interfere with my social life.

(49) I go out as often as I would like.

(50) I dedicate time to my hobbies and leisure as much as I would like.

(51) I meet my friends as much as I would like.

Personality

(52) I feel interested in food.

(53) I feel calm/peaceful.

(54) My personality has not changed.

Energy

(55) I feel rested most of the time.

(56) I am willing to do the things I want.

(57) I do not feel nausea during the day.

(58) I do not have hallucinations and delusions often.

(59) I do not get much sleep during the day.

(60) I do not get dizzy when I am lying/sitting and getting up.

Emotion/Humor

(61) I have confidence in myself.

(62) I often feel happy.

(63) I often feel peaceful.

(64) I feel excited about my future.

(65) I have no desire to be alone.

(66) I sleep and do not wake up many times during the night.

Is there any other aspect that affects you mentally/psychologically that has not been mentioned in this questionnaire?

Section C—Multidimensional Pain Inventory—MPI

The following statements refer to the impact of pain on your life. Please indicate how much you agree or disagree with each one. To do this, answer with one of the seven levels, ranging from 1 (a little) to 7 (a lot). They are classified progressively, that is, level 1 is the lowest, followed by level 2 and so on, up to level 7, which represents the highest value of the scale.

Did you feel pain in the last week? *
( ) Yes
( ) No

* If the answer is yes, the participant will answer the MPI. The participant will be instructed to assign only one score on a LIKERT scale ranging from 1 (a little) to 7 (a lot).

Severity of pain

(67) On average, how severe has your pain been during the last week?

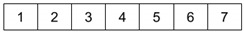

(68) How much suffering do you experience because of your pain?

(69) Estimate the level of your pain at this time.

Interference

(70) In general, how much does your pain interfere with your daily activities?

(71) From the moment your pain began, how much did your pain change your ability to work?

(72) How much did your pain change the amount of satisfaction or pleasure from your participation in social and leisure activities?

(73) How much did your pain change the amount of satisfaction or pleasure from your participation in family related activities?

(74) How much did your pain affect your relationship with your wife, family, or close people?

(75) How much did your pain change the amount of satisfaction or pleasure from your participation in job?

(76) How much did your pain affect your ability to do housework?

(77) How much did your pain change or interfere with your friendship with people other than your family?

(78) In general, how much did your pain affect your ability to participate in social activities?

Perceived life control

(79) During the last week, how much control did you feel you had over your life?

(80) During the last week, how much did you feel you were able to deal with everyday problems?

State of mood-affectivity

(81) Estimate your mood globally during the last week.

(82) During the last week, how irritable have you been?

(83) During the last week, how tense or anxious have you been?

## Appendix B. Validated Questionnaire—Multidimensional Assessment Questionnaire of Individuals with Parkinson’s Disease

Section A—Presentation of the Survey and Sample Description
I declare that I have Parkinson’s disease and that I am able to answer the questionnaire. *
( ) Yes
( ) No

I was diagnosed with Parkinson’s disease about: *
( ) Less than 2 years
( ) 2 to 4 years
( ) 4 to 6 years
( ) 6 to 8 years
( ) 8 to 10 years
( ) More than 10 years

The symbol * indicates that the participant must answer the question.

Section B—Individual Perception about Parkinson’s Disease
The following statements refer to some aspects of your daily life. Please indicate how much you agree or disagree with each statement. To do this, please answer with one of the five rating levels below:
0—Totally disagree
1—Disagree
2—Indifferent
3—Agree
4—Totally Agree

MODULE 1—Activities of daily living (ADL)
This module refers to activities of daily living, that is, tasks related to self-care and your ability to live independently.

(1) I hold the flatware without assistance.

(2) I handle the flatware without assistance.

(3) I take the flatware with the food up to my mouth without help and without spilling it.

(4) I chew the food without difficulty.

(5) I have no difficulty swallowing food.

(6) I serve liquid drinks without help and without spilling.

(7) I drink liquids without help and without spilling.

(8) I walk short distances without help.

(9) I walk long distances without help.

(10) I lie in bed without help.

(11) I get up from bed without help.

(12) I sit without help.

(13) I drive without difficulty.

(14) I climb stairs without help.

(15) I go down stairs without help.

(16) I dance without difficulty.

(17) I turn to bed at night without help.

(18) I get up from the car seat without help.

(19) I have balance when walking.

(20) I turn on the shower without help.

(21) I wash my body without help.

(22) I dry myself with the towel without help.

(23) I sit on the toilet without help.

(24) I get up from the toilet without help.

(25) I clean myself after evacuating without help.

(26) I brush my teeth without help.

(27) I wear a pant/skirt without help.

(28) I wear a shirt/blouse without help.

(29) I button my clothes without help.

(30) I open/close the zipper of my clothes without help.

(31) I put on my shoes without help.

(32) I tie the shoelaces without help.

(33) I take off my clothes to shower without help.

(34) I take my medicines without help.

(35) I make phone calls without help.

(36) I type messages on my cell phone without help.

(37) I write on a sheet of paper without help.

(38) I comb my hair without help.

(39) I use the television remote control without assistance.

(40) I hold a child in my arms without help.

(41) I speak clearly and without difficulty.

(42) I use Gillette without cutting myself.

(43) I read texts without help.

Is there any other activity in your daily life that you have a lot of difficulty or desire to do that has not been mentioned in this questionnaire?

MODULE 2—Cognitive aspects
This module refers to the cognitive aspects, that is, your feelings about life and society.

(46) I feel close and welcomed by other people.

(47) I am calm with other people.

(48) My physical condition does not interfere with my social life.

(49) I go out as often as I would like.

(50) I dedicate time to my hobbies and leisure as much as I would like.

(51) I meet my friends as much as I would like.

(53) I feel calm/peaceful.

(54) My personality has not changed.

(55) I feel rested most of the time.

(56) I am willing to do the things I want.

(61) I have confidence in myself.

(62) I often feel happy.

(63) I often feel peaceful.

(64) I feel excited about my future.

Is there any other aspect that affects you mentally/psychologically that has not been mentioned in this questionnaire?

Section C—Multidimensional Pain Inventory—MPI
The following statements refer to the impact of pain on your life. Please indicate how much you agree or disagree with each one. To do this, answer with one of the seven levels, ranging from 1 (a little) to 7 (a lot). They are classified progressively, that is, level 1 is the lowest, followed by level 2 and so on, up to level 7, which represents the highest value of the scale.

Did you feel pain in the last week? *
( ) Yes
( ) No

* If the answer is yes, the participant will answer the MPI. The participant will be instructed to assign only one score on a LIKERT scale ranging from 1 (a little) to 7 (a lot).

(67) On average, how severe has your pain been during the last week?

(68) How much suffering do you experience because of your pain?

(69) Estimate the level of your pain at this time.

(70) In general, how much does your pain interfere with your daily activities?

(71) From the moment your pain began, how much did your pain change your ability to work?

(72) How much did your pain change the amount of satisfaction or pleasure from your participation in social and leisure activities?

(73) How much did your pain change the amount of satisfaction or pleasure from your participation in family related activities?

(74) How much did your pain affect your relationship with your wife, family, or close people?

(75) How much did your pain change the amount of satisfaction or pleasure from your participation in job?

(76) How much did your pain affect your ability to do housework?

(77) How much did your pain change or interfere with your friendship with people other than your family?

(78) In general, how much did your pain affect your ability to participate in social activities?

(82) During the last week, how irritable have you been?

(83) During the last week, how tense or anxious have you been?
